# Fabrication of spent FCC catalyst composites by loaded V_2_O_5_ and TiO_2_ and their comparative photocatalytic activities

**DOI:** 10.1038/s41598-019-47155-y

**Published:** 2019-07-31

**Authors:** Jiasheng Xu, Te Zhang

**Affiliations:** 1 College of Chemistry, Chemical Engineering and Environmental Engineering, Liaoning Shihua University, Fushun, 113001 P.R. China; 2 Liaoning Province Key Laboratory for Synthesis and Application of Functional Compounds, College of Chemistry and Chemical Engineering, Bohai University, Jinzhou, 121013 P.R. China

**Keywords:** Structural properties, Photocatalysis

## Abstract

The spent fluid catalytic cracking catalyst (FCC) has been loaded with different content of V_2_O_5_ and TiO_2_ through a modified-impregnation method. X-ray Diffraction (XRD), ultraviolet-visible spectrophotometry (UV-Vis), Scanning Electron Microscope (SEM), and Fourier Transform Infrared spectroscopy (FT-IR) are used to characterize the structure and morphology of these samples. Their photocatalytic activity was evaluated by degradation of methylene blue (MB) solution under 300 W Xenon lamp irradiation. The interplanar spacing of the zeolite Y (111) plane is affected by the amount of the loaded V_2_O_5_ on spent FCC catalyst. The (111) plane of spent FCC catalyst loaded with V_2_O_5_ and TiO_2_ sample is 1.404 nm, which is higher than that of the zeolite Y (1.395 nm). The amount of adsorption of MB and the photocatalytic activity for the degradation increased with increasing the interplanar spacing of the (111) plane of sample. We fabricated of spent FCC catalyst composites by loaded V_2_O_5_ and TiO_2_, which effectively solved the spent FCC catalyst disposal problem. The efficiency of the developed sample provides a potentially economical way of degrading MB.

## Introduction

In the petroleum refining industry, the fluid catalytic cracking (FCC) process is one of the most important processes^1–8^. The FCC catalyst deactivates with time and when the activity of the catalyst declines below the unacceptable level, it is usually disposed as hazardous waste^9^. The Ni, V, Fe and coke from petroleum crude oil are deposited onto the surface of the FCC catalyst particle^10,11^, and these impurities are even embedded in the zeolite Y framework of the FCC catalyst. Every year, more than 160,000 tons of spent catalyst are generated in the petrochemical industry^12^. The spent FCC catalyst has been listed as HW50 type hazardous waste, which was included in the Chinese National Hazardous Waste List (2016 version)^13^. The treatment method of the spent FCC catalyst is primarily the landfill method^14^, which cause serious environmental pollution and land consumption. The spent FCC catalyst is used in cement production and as a cement additive. It makes cement more suitable for practical applications^15^. A few works use spent FCC catalyst to prepare the potential anti-corrosive and anti-biofouling materials, which indicated high corrosion inhibition efficiency^16^.

The zeolite Y framework, the pore structure and the surface area structure for the spent FCC catalyst are almost unchanged, which still has typical useful value used to support photocatalysis, although the spent FCC catalyst loses its catalytic activity^17^. The treatment and recycling of the spent FCC catalyst through other valuable processes is attractive from environmental and economic points of view. The zeolite Y framework provides high surface area and adsorbent capacity. In recent years, the spent FCC catalyst as supporter for TiO_2_ is utilized for the photodegradation of dye solution^18^. TiO_2_ is activated by UV irradiation due to its large band gap (3.2 eV)^19^. V_2_O_5_ has band edges at E_CB_ = 0.47 eV and E_VB_ = 2.73 eV, which can match well with TiO_2_ (E_CB_ = −0.1 eV, E_VB_ = 3.1 eV) to form a photocatalytic system^20,21^. These compounds display a high photocatalytic performance for degradation of the dye solution. The advantage of photocatalytic degradation of MB by use the spent FCC catalyst to supported TiO_2_ and V_2_O_5_ was the modulation of properties of the semiconductors by changing the valance and conduction bands in order to be activated under light irradiation^22^. It can induce a synergistic effect; they interact to generate a composite material with a favorably high surface area and high catalytic activity^23^.

In this work, the V/FCC, Ti/FCC, V-Ti/FCC and V-Ti-2/FCC samples are prepared by a modified-impregnation method. The samples are characterized by XRD, SEM, UV-Vis and FT-IR. The photocatalytic performance and adsorptive behavior of these samples are tested. The activity of the obtained V/FCC, Ti/FCC, V-Ti/FCC and V-Ti-2/FCC samples are evaluated by studying the degradation of MB solution. The degradation of MB by use of V/FCC, Ti/FCC, V-Ti/FCC and V-Ti-2/FCC samples are ~26%, ~36%, ~75% and ~96%, respectively. These results indicated that V-Ti-2/FCC composites exhibited good photocatalytic activity.

## Result and Discussions

### Characterization of prepared photocatalysts

**Figure 1:**
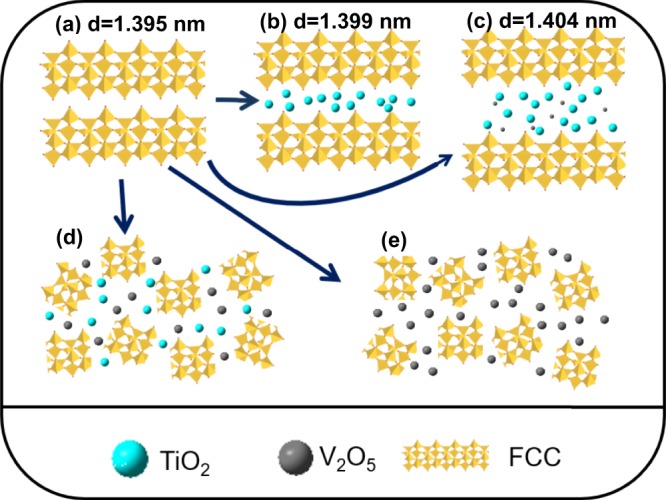
Schematic illustration of the fabrication of spent FCC catalyst composites. (**a**) Spent FCC catalyst, (**b**) Ti/FCC, (**c**) V-Ti-2/FCC, (**d**) V-Ti/FCC and (**e**) V/FCC.

The V/FCC, Ti/FCC, V-Ti/FCC and V-Ti-2/FCC samples are rationally designed and fabricated by a modified-impregnation method, which can be directly employed as photocatalysts for MB solution degradation. Figure 1 shows the schematic illustration of the spent FCC catalyst, which are loaded by V_2_O_5_ and TiO_2_. This schematic illustration can be helpful in understanding photocatalytic activities of these samples.

**Figure 2:**
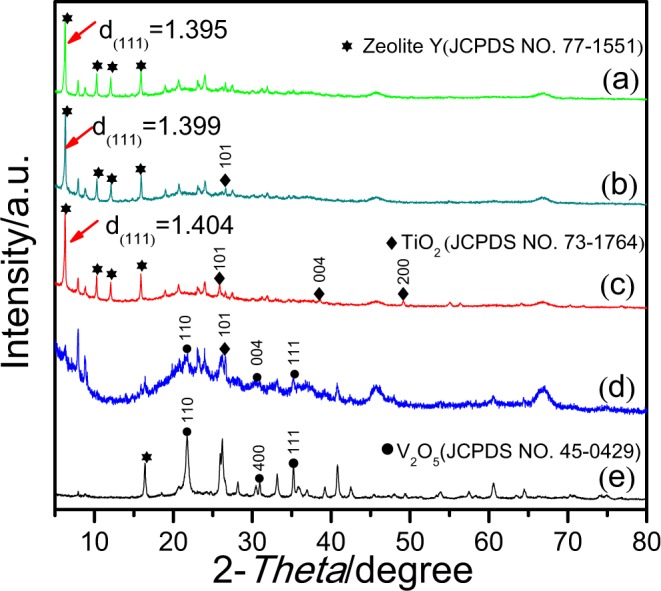
XRD patterns of these samples. (**a**) Spent FCC catalyst, (**b**) Ti/FCC, (**c**) V-Ti-2/FCC, (**d**) V-Ti/FCC and (**e**) V/FCC. The peaks generated by zeolite Y are marked with hexagram (JPDS card No. 77–1551); the peaks generated by TiO_2_ are marked with rhombus (JPDS card No. 73–1764); the peaks generated by V_2_O_5_ are marked with dot (JPDS card No. 45–0429).

Figure 1a shows the (111) plane of the spent FCC catalyst (zeolite Y). The interplanar spacing of the zeolite Y is 1.395 nm. Figure 1b shows the (111) plane of the Ti/FCC sample, with interplanar spacing at 1.399 nm. Figure 1c shows the (111) plane of the V-Ti-2/FCC sample, with interplanar spacing at 1.404 nm. It is clear to see that the interplanar spacing is increased, once the different content of V_2_O_5_ and TiO_2_ is loaded on spent FCC catalyst. As shown in the XRD patterns (Fig. 2d–e), there are no reflections of the (111) planes of the V-Ti/FCC and V/FCC samples; the (111) plane of V-Ti/FCC and V/FCC sample have been collapsed. V_2_O_5_ attacks the zeolite Y, which make the interplanar spacing change^24^. The interplanar spacing of the (111) plane of zeolite Y can be changed during this modified-impregnation process. The V-Ti-2/FCC sample has the highest interplanar spacing of the (111) plane, indication that the V-Ti-2/FCC sample can adsorb more methylene blue.

The XRD patterns (2*θ* range from 5° to 80°) of the spent FCC catalyst, Ti/FCC, V-Ti-2/FCC, V-Ti/FCC and V/FCC samples are shown in Fig. 2. The diffraction peaks located at 6.3°, 10.3°, 12.1° and 15.91° are the characteristic peaks of the spent FCC catalyst sample (Fig. 2a), which match well with the standard zeolite Y phase (JPDS card No. 75–1551). These diffraction peaks correspond to the reflections of the (111), (220), (311) and (331) planes of the zeolite Y arrays, respectively. In Fig. 2b, the four diffraction peaks located at 6.3°, 10.3°, 12.1° and 15.9° are the characteristic peaks of the FCC, which correspond to the (111), (220), (311) and (331) planes of the standard zeolite Y phase (JPDS card No. 75–1551), respectively. One diffraction peak located at 26.5°is the characteristic peak of the TiO_2_, which correspond to the (101) plane of the standard TiO_2_ phase (JPDS card No. 73–1764). In Fig. 2c, four diffraction peaks located at 6.3°, 10.3°, 12.1° and 15.9° are the characteristic peaks of the zeolite Y, which correspond to the (111), (220), (311) and (331) planes of the zeolite Y phase (JPDS card No. 75–1551), respectively. Three diffraction peaks located at 26.5°, 35.4° and 40.1° are the characteristic peaks of the TiO_2_, which correspond to the (101), (004) and (200) planes of the TiO_2_ phase (JPDS card No. 73–1764), respectively. In Fig. 2d, three diffraction peaks located at 24.1°, 30.9° and 34.0° are characteristic peaks of the V_2_O_5_, which correspond to the (110), (400) and (111) planes of the V_2_O_5_ phase (JPDS card No. 45–0429), respectively. One diffraction peak located at 26.5° is the characteristic peak of the TiO_2_, which corresponds to the (101) plane of TiO_2_ phase (JPDS card No. 73–1764). In Fig. 2e, three diffraction peaks correspond to the (110), (400) and (111) planes of the V_2_O_5_ phase (JPDS card No. 45–0429), respectively. One diffraction peak located at 15.9° is the characteristic peak of zeolite Y, which corresponds to the (331) plane of zeolite Y phase (JPDS card No. 77–1551).

**Figure 3:**
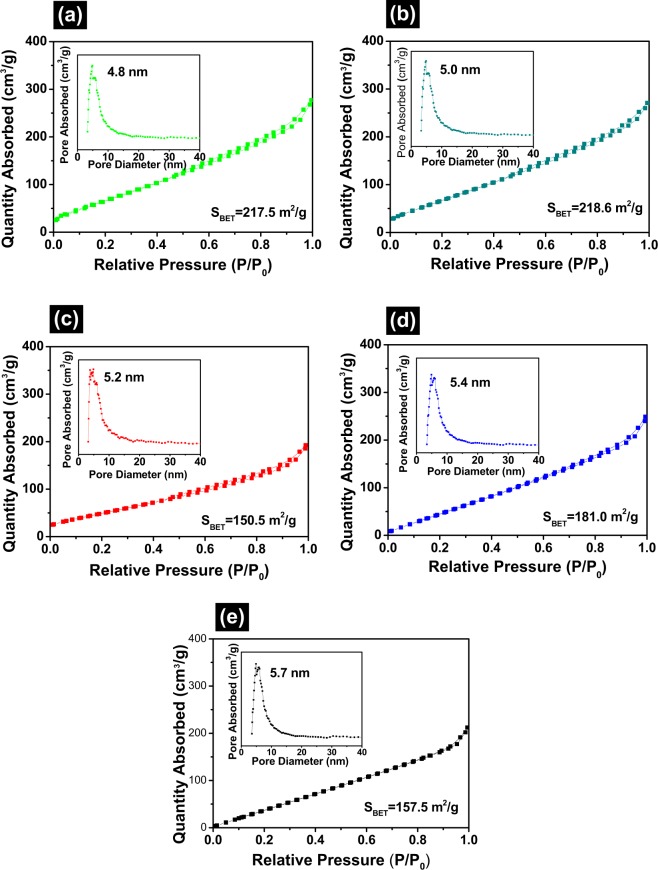
Brunauer-Emmett-Teller (BET) nitrogen adsorption and desorption isotherms of (**a**) spent FCC catalyst, (**b**) Ti/FCC, (**c**) V-Ti-2/FCC, (**d**) V-Ti/FCC and (**e**) V/FCC, inset shows its pore size distribution curve. (Test condition: Nitrogen adsorption and desorption isotherm were measured using nitrogen (99.999%) and helium at liquid nitrogen baths of 77 K).

Specific surface areas generally influence the adsorption and catalytic performance of photocatalysts. Nitrogen adsorption-desorption isotherms and corresponding pore size distribution curves of spent FCC catalyst, V/FCC, Ti/FCC, V-Ti/FCC and V-Ti-2/FCC samples were shown in Fig. 3a–e, respectively. The isotherms of all samples were of type-IV with a H3 hysteresis loop in the relative pressure (P/P0) indicating the presence of a macroporous structure which were representative of mesoporous materials according to the IUPAC classification. The pore-size distribution of spent FCC catalyst, V/FCC, Ti/FCC, V-Ti/FCC and V-Ti-2/FCC samples was largest around 4.8 nm, 5.7 nm, 5.0 nm, 5.4 nm and 5.2 nm, respectively. As shown in Fig. 3a–e, BET specific surface areas of spent FCC catalyst, V/FCC, Ti/FCC, V-Ti/FCC and V-Ti-2/FCC samples were 217.5, 157.5, 218.6, 181.0 and 150.0 m^2^/g, respectively. The V_2_O_5_ and TiO_2_ loading decreased the surface area (S_BET_) as it can be seen from the data. The pore size of the V/FCC, Ti/FCC, V-Ti/FCC and V-Ti-2/FCC samples were increased significantly, indicating that the deposition of V_2_O_5_ and TiO_2_ onto the spent FCC catalyst surface causes changing of the zeolite Y pore.

**Figure 4:**
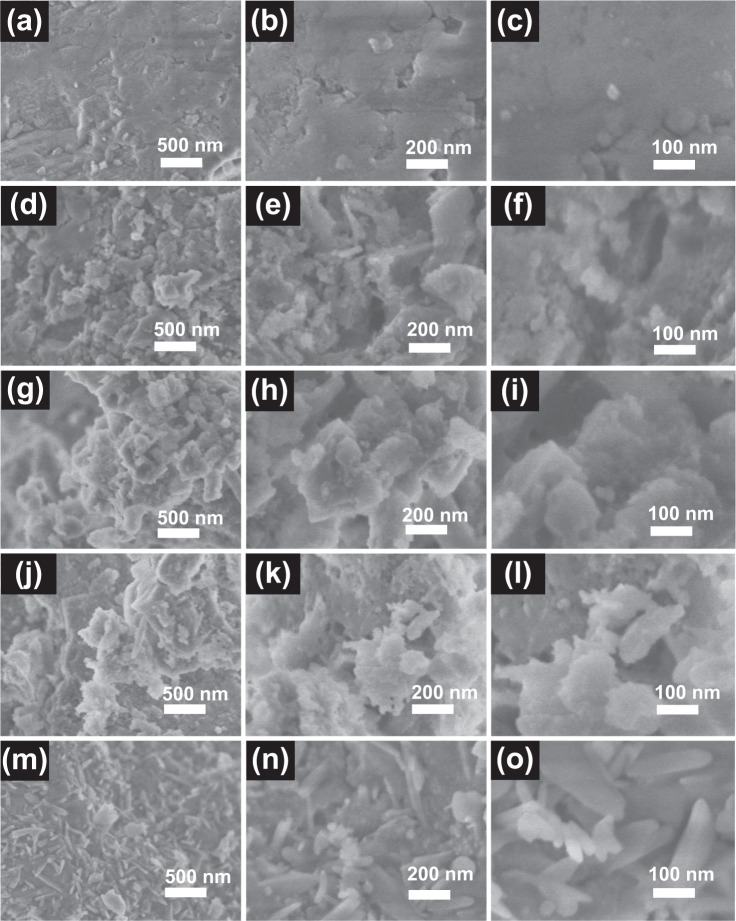
SEM images of the samples FCC, Ti/FCC, V-Ti-2/FCC, V-Ti/FCC and V/FCC with different morphologies by altering loaded different content of the V_2_O_5_ or TiO_2_. (**a**–**c**) The different magnifications SEM images of the FCC sample, scale bars = 500 nm, 200 nm and 100 nm, respectively; (**d**–**f**) the Ti/FCC sample, scale bars = 500 nm, 200 nm and 100 nm, respectively; (**g**–**i**) the V-Ti-2/FCC sample, scale bars = 500 nm, 200 nm and 100 nm, respectively; (**j**–**l**) the V-Ti/FCC sample, scale bars = 500 nm, 200 nm and 100 nm, respectively; (**m**–**o**) the V/FCC sample, scale bars = 500 nm, 200 nm and 100 nm, respectively.

Figure 4 shows the SEM images of the spent FCC catalyst, V/FCC, Ti/FCC, V-Ti/FCC and V-Ti-2/FCC samples. These SEM images show the different morphological structures. The morphologies and nanostructures of the original sample (spent FCC catalyst) are shown in Fig. 4a–c. The low-magnification SEM image shows the morphology of the spent FCC catalyst surface. The high-magnification SEM images of the spent FCC catalyst sample shows the surface, which appears to break and collapse^10^. The framework (large specific surface area and pore volume) of the zeolite Y is basically intact, which is favorable to support the nanostructure of TiO_2_ and V_2_O_5_.

The Ti/FCC sample is obtained by loaded TiO_2_ on the spent FCC catalyst. Figure 4d–f show the SEM images of the Ti/FCC sample. The low-magnification SEM image (Fig. 4d) of the Ti/FCC sample reveals that the smooth surface of the spent FCC catalyst is covered with TiO_2_ particles. From the SEM images (Fig. 4e), it is evident that the surface of spent FCC sample is covered with TiO_2_ particles. They are either deposited on the surface of the spent FCC sample. The high-magnification SEM image (Fig. 4f) of the Ti/FCC sample reveals that the TiO_2_ tightly adhered to the spent FCC catalyst to form the heterojunction structure.

The different morphological V-Ti-2/FCC sample images are obtained by loaded TiO_2_ and V_2_O_5_ particles on the surface of the spent FCC catalyst. Figure 4g–i show the morphologies and nanostructures of the V-Ti-2/FCC sample. The low-magnification SEM image (Fig. 4g) shows that the smooth FCC catalyst is covered with TiO_2_ and small amounts of V_2_O_5_ particles. Figure 4h,i display a high-magnification SEM image of the V-Ti-2/FCC sample. The spent FCC catalyst surface is covered with TiO_2_ and V_2_O_5_.

The morphologies and nanostructures of the V-Ti/FCC sample are shown in Fig. 4j,k. The high-magnification SEM images show that the V_2_O_5_ and TiO_2_ grow almost in clusters on the spent FCC catalyst surface. The morphologies and nanostructures of the V/FCC sample are shown in Fig. 4m–o. The low-magnification SEM image (Fig. 4m) shows the V_2_O_5_ grown on the spent FCC catalyst surface. From Fig. 4n,o, we can see that some V_2_O_5_ particles in sizes of 50–300 nm are deposited on their surfaces.

**Figure 5:**
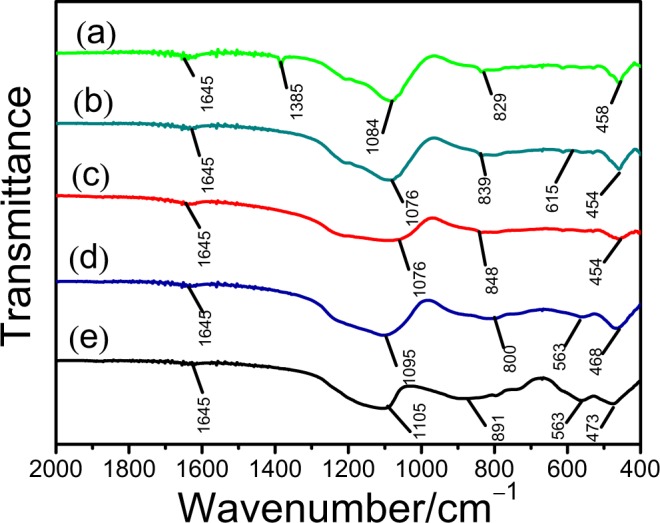
FT-IR spectra of samples. (**a**) Spent FCC catalyst, (**b**) Ti/FCC, (**c**) V-Ti-2/FCC, (**d**) V-Ti/FCC and (**e**) V/FCC.

FT-IR spectra of the Ti/FCC, V-Ti-2/FCC, V-Ti/FCC and V/FCC samples are shown in Fig. 5. In Fig. 5a-e, there is an absorption band at 1645 cm^−1^, which is attributed to the distorted OH group stretching vibration in the zeolite Y^25,26^. In Fig. 5a, the strong peak at 1084 cm^−1^ corresponds to the Si–O–Si(Al) stretching vibration^27^. The bands at 829 cm^−1^ and 458 cm^−1^ are attributed to the Si(Al)–O stretching vibration^26^. In Fig. 5b, the characteristic stretching vibration of Si–O–Si shifts from 1084 cm^−1^ to 1076 cm^−1^, which is due to the interrelationship of TiO_2_ and zeolite Y. There is no band of the Si–O–Ti antisymmetric stretching vibration in the region near at 960 cm^−1^. The band at 454 cm^−1^ is attributed to the Ti–O–Ti stretching vibration. It is indicated that the TiO_2_ compound is deposited on the spent FCC catalyst^28^. In Fig. 5c, the band at 1076 cm^−1^ is attributed to the Si–O–Si(Al) stretching vibration. The band at 848 cm^−1^ originates from the Si–O stretching vibration. The band at 454 cm^−1^ is attributed to the stretching vibration of Ti–O–Ti. In Fig. 5d, the band at 1095 cm^−1^ is attributed to the stretching vibration of Si–O–Si. The band at 563 cm^−1^ is assigned to the stretching vibration of V–O–V. The band at 473 cm^−1^ is attributed to the Ti–O–Ti stretching vibration. In Fig. 5e, the band at 1105 cm^−1^ is attributed to the Si–O–Si stretching vibration. The band at 891 cm^−1^ and 563 cm^−1^ are attributed to V–O–V stretching vibration.

**Figure 6:**
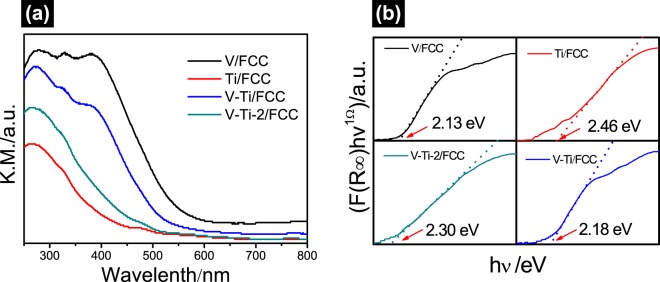
(**a**) The UV-Vis reflection spectra of V/FCC, Ti/FCC, V-Ti-2/FCC and V-Ti/FCC samples. (**b**) The transformations of Kubelka-Munk function of V/FCC, Ti/FCC, V-Ti/FCC and V-Ti-2/FCC samples.

The optical absorption characteristics of the V/FCC, Ti/FCC, V-Ti/FCC and V-Ti-2/FCC samples are tested, and results are shown in Fig. 6a. It is obvious that recorded spectra of these samples show almost similar shape and differences in intensities. These samples have different positions of the absorption edge. It can be observed that the light absorption of the Ti/FCC sample is the lowest of all the samples. The absorption threshold of Ti/FCC is about 420 nm. The absorption threshold of V/FCC is about 563 nm. The UV–Vis spectrum of the V-Ti-2/FCC sample indicates that it absorbs light with a wavelength less than 460 nm. The absorption threshold of the V-Ti/FCC sample is about 520 nm. As shown in Fig. S1, the absorption thresholds of the spent FCC catalyst, V_2_O_5_ and TiO_2_ are about 493 nm, 582 nm and 406 nm, respectively. It can be observed that light absorption in the visible region (300–800 nm) of the V-Ti/FCC composites is higher than that of V-Ti-2/FCC.

Basing on the absorbance spectra, the band gap energies of the V/FCC, Ti/FCC, V-Ti-2/FCC and V-Ti/FCC samples are estimated from the Kubelka-Munk function. Figure 6b shows band gaps of these samples, which is determined by an intercept of the tangent line to the X-axis. These show the spectrum of energy versus^29,30^ [*F*(*R*_*∞*_) *hv*]^*n*^. The band gap energy and absorption edge of V/FCC, Ti/FCC, V-Ti-2/FCC and V-Ti/FCC samples are 2.13, 2.46, 2.18 and 2.30 eV, respectively. The widest band gap energy (around 2.46 eV) is shown by the Ti/FCC sample. TiO_2_ shows the band gap is 3.25 eV. The band gap energy of the V-Ti/FCC and V-Ti-2/FCC samples are estimated to be 2.18 eV and 2.30 eV^31–33^. The partial absorption in the visible range is determined by the band gap energy value, which shows that these samples have potential photocatalytic activity. The band gap energy of the V_2_O_5_ is shown in Fig. S2. V_2_O_5_ has suitable band edges (E_VB_ = 2.73 eV, E_CB_ = 0.47 eV), which can correspond with TiO_2_ (E_VB_ = 3.1 eV, E_CB_ = −0.1 eV) to form a photocatalytic system^34,35^. The V/FCC has been found possess a narrow band gap in comparison to that of the other samples. The narrow band gap is a good candidate, capable of capturing visible light.

**Figure 7:**
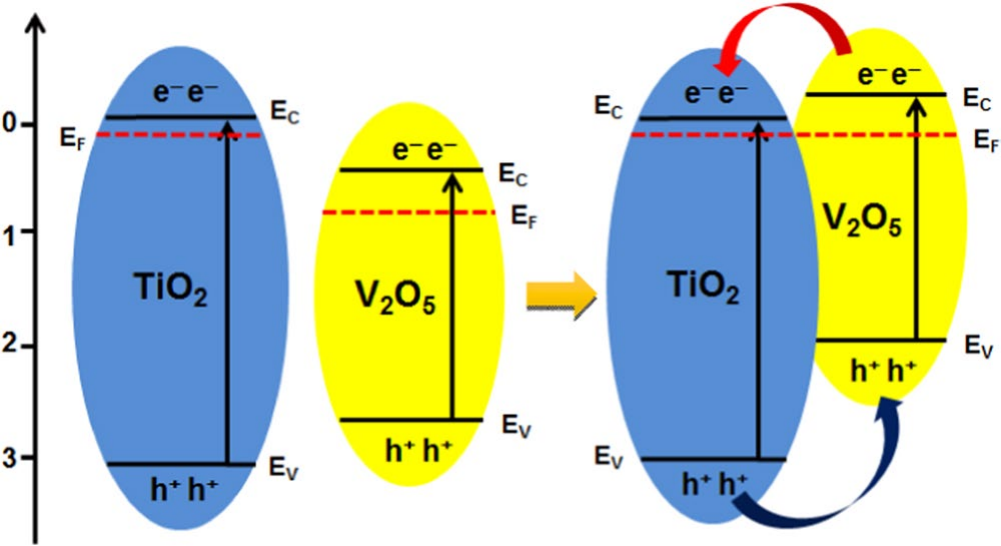
Schematic of energy bands matching and electron-hole pair separation of the V-Ti-2/FCC sample.

The following figures clarify the photocatalytic mechanism of the V-Ti-2/FCC sample based on the electronic structures. Figure 7 shows the illustration of interparticle electron transfer behavior. The conduction band (CB) energy position of TiO_2_ is −0.1 eV. The CB energy of V_2_O_5_ is 0.47 eV. When the V_2_O_5_ and TiO_2_ form a heterostructure, the Fermi energy of the two materials has to be the same^32,35–37^. As a result, the CB and VB of V_2_O_5_ move upward. In the photocatalysis reaction process, V_2_O_5_ and TiO_2_ form a heterostructure. The photo-generated electrons in CB of V_2_O_5_ migrate to TiO_2_ while the photo-generated holes transfer from the VB of TiO_2_ to that of V_2_O_5_. The photo-induced electrons could rapidly transfer from the CB of the V_2_O_5_ to that of the TiO_2_, which significantly promotoes the separation of photo-induced electrons and holes.

### Evaluation of photocatalytic activity

**Figure 8:**
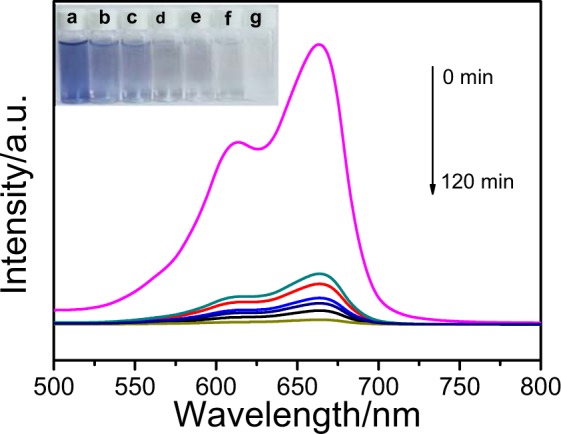
Spectral patterns of the methylene blue solution during the photocatalytic reduction process in the presence of the V-Ti-2/FCC sample for 120 min. (inset shows the photographs of methylene blue solution: the 0 min, 20 min, 40 min, 60 min, 80 min, 100 min and 120 min, which corresponds with (**a**–**g**) respectively).

The photocatalytic activity of the V-Ti-2/FCC sample is analyzed by photocatalytic degradation of the MB solution. From Fig. 8, the peak at ~664 nm is the absorption characteristic of the MB. With the photocatalytic time extending, the absorption peak intensity of the MB decreases rapidly. It is found that the concentration of MB rapidly decreases with the increase of irradiation time. The color of the MB solution almost disappears after 120 min, which suggests the complete destruction of the methylene blue conjugated structure. The inset in Fig. 7 shows the photographs of the MB solution: 0, 20, 40, 60, 80, 100 and 120 min, which corresponds with (a), (b), (c), (d), (e), (f) and (g), respectively. It can be seen that 96% of the MB solution has already been degraded by the V-Ti-2/FCC sample, after 120 min of irradiation.

The V-Ti-2/FCC sample generated hole (*h*^*+*^) and electrons (*e*^−^) under the light irradiation^38^. The detail process can be expressed as follows 1 and 2:

1 $${\rm{V}}-\mathrm{Ti}-2/{\rm{FCC}}+hv\to {h}^{+}+{e}^{-}$$

2 $${\rm{MB}}+hv\to {{\rm{MB}}}_{\ast }\to {\rm{intermediate}}\to {{\rm{CO}}}_{2}+{{\rm{H}}}_{2}{\rm{O}}+{\rm{other}}\,{\rm{groups}}$$

**Figure 9:**
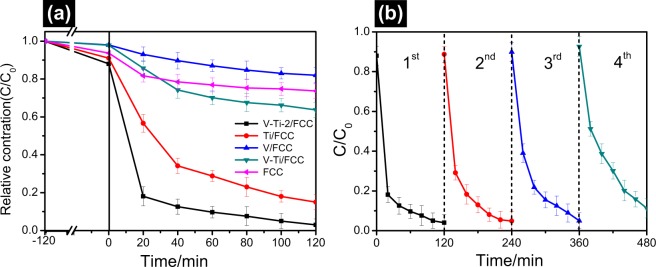
(**a**) Photocatalytic degradation of methylene blue on the spent FCC catalyst, V/FCC, Ti/FCC, V-Ti/FCC and V-Ti-2/FCC samples, respectively (*C*_0_ = 10 mg/L, catalyst concentration = 1.0 g/L). (**b**) Methylene blue degradation performance in four cycles for the V-Ti-2/FCC.

The photocatalytic degradation performances of the spent FCC catalyst, V/FCC, Ti/FCC, V-Ti/FCC and V-Ti-2/FCC samples in the degradation of MB solution are monitored. Figure 9a shows the degradation results of these samples. The activity of the spent FCC catalyst, V/FCC, Ti/FCC, V-Ti/FCC and V-Ti-2/FCC samples are evaluated. The absorption spectra in the presence of the as-prepared samples indicate that the spent FCC catalyst, V/FCC, Ti/FCC, V-Ti/FCC and V-Ti-2/FCC samples adsorbed ~3%, ~3%, ~7%, ~11% and ~14% of MB, respectively. The dye remaining percentage for the presence of the spent FCC catalyst, V/FCC, Ti/FCC, V-Ti/FCC and V-Ti-2/FCC samples are ~19%, ~26%, ~36%, ~75% and ~96%, respectively, after light irradiation for 120 min. The optimal decomposition ratio of MB over the V-Ti-2/FCC sample is 96% after light irradiation for 120 min. In contrast, under the same conditions, the MB is degraded only about 20% over the V-Ti/FCC sample. In all samples, the V-Ti-2/FCC sample displays a higher photocatalytic activity than other samples. All the above photocatalytic results indicate that the V-Ti-2/FCC photocatalyst displays excellent photocatalytic performance.

The stable reusability of V-Ti-2/FCC sample was investigated by a four-cycle degradation of methylene blue under identical conditions. The reusability is vital factor influencing practical application of V-Ti-2/FCC sample in dye wastewater treatment. As shown in Fig. 9b, after four cycle experiments in light irradiation, the photocatalytic degradation efficiency barely changes, with the degradation rate of about 89% during the four experiment. These results demonstrate the feasibility of recovery reuse of the V-Ti-2/FCC sample. Figure S3 shows the performance of the recycled V-Ti-2/FCC sample for the methylene blue photocatalytic degradation. It was found that the methylene blue degradation efficiency of V-Ti-2/FCC was still up to ~96%, ~95%, ~95% and ~89%, respectively.

## Conclusions

In summary, we have successfully fabricated the spent FCC catalyst composites by loaded V_2_O_5_ and TiO_2_ via a modified-impregnation method, which are applied as photocatalysts to degrade the methylene blue solution. The photo-induced electrons from the CB of V_2_O_5_ rapidly transfer to the CB of the TiO_2_ during the photocatalytic reaction process, which promotes the separation of photo-induced electrons on the holes. The V-Ti-2/FCC sample can significantly improve the photocatalytic activity *via* increasing the number of photoinduced charge carriers at the interface of the V_2_O_5_ and TiO_2_ structure. The optimal decomposition ratio of methylene blue over V-Ti-2/FCC sample is 96% under light irradiation for 120 min. The degradation ratios of methylene blue over the V/FCC, Ti/FCC and V-Ti/FCC samples were ~26%, ~36% and ~75%, respectively. This investigation may provide guidance for the treatment of the spent FCC catalysts and these spent FCC catalyst composites can serve as potential photocatalytic materials, which indicates their potential application in the field of dye solution degradation.

## Experimental Section

### Fabrication of composites

The spent FCC catalyst composites have been fabricated with different content of V_2_O_5_ and TiO_2_, using the spent FCC catalyst as support. The first one was fabricated by V_2_O_5_, where 2 g of spent FCC catalyst powder was impregnated with 0.2 g of V_2_O_5_ and 3 mL H_2_O_2_. The mixture was grinded for 2 h. The resultant sample was denoted as V/FCC. The second one was fabricated by tetrabutyl titanate impregnation, and the fabrication process is the same to that of the first one except for the addition of 2 mL of tetrabutyl titanate. The resultant sample was denoted as Ti/FCC. The third one was fabricated by V_2_O_5_ and tetrabutyl titanate, and the fabrication process is the same to that of the first one except for the addition of 0.1 g of V_2_O_5_ and 1 mL tetrabutyl titanate. The resultant sample was denoted as V-Ti/FCC. The fourth one was fabricated by V_2_O_5_ and tetrabutyl titanate, and the fabrication process is the same to that of the first one except for the addition of 0.02 g of V_2_O_5_ and 0.18 mL tetrabutyl titanate. The resultant sample was denoted as V-Ti-2/FCC. All the samples were calcined at 800 °C for 4 hours.

### Characterizations

The crystalline structure of these samples were characterized by the powder XRD (Rigaku RAD-3C). Data was collected in the Cu Kα, *λ* = 1.5405 Å, 35 kV, 20 mA, 2-*Theta* angles (5°–80°), and the scan rate was 10° min^−1^. The morphology of these samples was measured using the scanning electron microscope with a JEOL S-4800 of FE-SEM under the condition of 3.0 kV operating voltage. Gas adsorption behavior and Brunauer–Emmett–Telle (BET) surface area of the samples were determined using a Micrometrics (Tristar, 3000) instrument in nitrogen atmosphere. Diffuse Reflectance UV-vis was recorded, with select wavelength coverage from 200 nm to 800 nm. FT-IR spectra were recorded by a spectrometer (Thermo Scientific^TM^ Nicolet is 5 FT-IR Spectrometer) at room temperature in the region of 400–4000 cm^−1^. The concentrations of the methylene blue relative to the photocatalysis time were tested by UV-Vis spectrophotometer.

### Photocatalytic tests

Methylene blue was used as a model to investigate the adsorption and photocatalytic activities of these samples (FCC, V/FCC, Ti/FCC, V-Ti/FCC and V-Ti-2/FCC). The concentration of MB solution was determined by UV–vis spectrophotometer at 20 min. The photocatalytic degradations of these catalysts for methylene blue solution were performed in a 1000 mL reactor. The solution was irradiated using a Xenon lamp (300 W) equipped with a filter cutoff of 400 nm.

The adsorption–desorption equilibrium was determined by measuring the dye concentration for 2 h. There was no light irradiation during the time that 0.2 g of catalyst was added in a 200 mL MB solution. It was stirred in darkness to obtain a good dispersion and reach adsorption-desorption equilibrium between the methylene blue and the catalyst surface. Then the solution was irradiated under a 300 W Xenon lamp for 2 h. The concentration of the remaining MB solution was analyzed every 20 min. The photodegradation efficiency (*A*) was given by formula (3).

3 $$A=\frac{{C}_{0}-C}{{C}_{0}}$$

The *C*_*0*_ is the initial methylene blue solution concentration, and the *C* is the concentration of methylene blue solution.

## Supplementary information

Supplementary Information
