# Supplementary material for: Fabrication of spent FCC catalyst composites by loaded V2O5 and TiO2 and their comparative photocatalytic activities
Source: Sci Rep. 2019 Jul 31;9:11099. doi: 10.1038/s41598-019-47155-y (PMC6668428; doi:10.1038/s41598-019-47155-y)
Supplement: Supplementary file 1 — Supplementary Information [file 41598_2019_47155_MOESM1_ESM.docx]

**Supplementary Information**

**Fabrication of** **spent FCC catalyst composites by loaded V_2_O_5_ and** **TiO_2_ and** **their comparative photocatalytic activities**

Jiasheng Xu*^1,2^*, Te Zhang*^2^*

*^1^College of Chemistry, Chemical Engineering and Environmental Engineering, Liaoning Shihua University, Fushun, 113001, P.R. China.*

*^2^Liaoning Province Key Laboratory for Synthesis and Application of Functional Compounds, College of Chemistry and Chemical Engineering, Bohai University, Jinzhou 121013, P.R. China.*

*Correspondence and requests for materials should be addressed to J. X. (email:* [*jiashengxu@bhu.edu.cn*](mailto:liuxy@jlu.edu.cn)*)*

**Figure S1.** The UV-Vis reflection spectra of the spent FCC catalyst, V_2_O_5_ and TiO_2_ samples.

**Figure S2.** The transformation of Kubelka-Munk function of V_2_O_5_.

**Figure S3.** Methylene blue degradation performance in four cycles for the V-Ti-2/FCC.
